# ‘Well-Track’: Fitbit Based Sleep and Physical Activity Intervention for Severe Mental Illness (SMI) Patients

**DOI:** 10.1192/bjo.2024.155

**Published:** 2024-08-01

**Authors:** Chris Griffiths, Alice Sheldon, Gerry Smith, Kate Walker, Harmony Jiang

**Affiliations:** Northamptonshire Healthcare NHS Foundation Trust, Northampton, United Kingdom

## Abstract

**Aims:**

Compared with general population average, people experiencing severe mental illness (SMI) have lower levels of physical activity, high levels of sedation, and more sleep problems (Soundy et al. 2013; Vancampfort et al. 2015). This is linked to symptoms of depression, lower wellbeing, hopelessness, lower quality of life and physical health conditions, such as: cardiovascular disease (CVD), stroke, hypertension, osteoarthritis, diabetes, and chronic obstructive pulmonary disease (COPD) (Rhodes et al. 2017; Schuch et al. 2017). Engaging in physical activity improves quality of life, psychotic symptomatology, cognition, functioning and physical health (Mittal et al. 2017). Improved sleep is associated with enhanced social interaction, feeling energised, and improved engagement in activities (Waite et al. 2016). NHS Long-Term Plan (2022) is to ensure that at least 80% of people with SMI receive an annual 12 point physical health check. Professor Helen Lester stated: 'Don't just screen, intervene'. There is an urgent need to provide interventions that improve the healthy lifestyles of people with SMI, but there is a lack of suitable and effective interventions. To be effective, interventions need to be individualised (Griffiths et al. 2021).

**Intervention and aims**

Well-Track is the provision of a Fitbit and its software apps, sleep hygiene and physical activity guidance, motivational interviewing, workbook goal setting through three sessions with a health coach. Aim was to improve sleep, physical activity, wellbeing, and healthy lifestyles.

**Methods:**

Outcome measure data collection from baseline to 3 and 6 week follow-ups. Change in sleep quality and wellbeing were assessed in 50 participants, and participant feedback was obtained.

**Results:**

Improvements were found in sleep quality and wellbeing. Most patients attended all three sessions and actively used the Fitbit and its software apps, guidance and workbook to set goals and to make positive changes to their lifestyle and daily routines to improve motivation, quality of sleep, and level of physical activity.

**Conclusion:**

Healthy effective sleep and physical activity/exercise are important to SMI patients’ wellbeing and mental and physical health. A health coach successfully and fully integrated the Well-Track intervention into routine service provision. The intervention was beneficial, relatively easy and low cost to implement, and well-liked by patients and staff; and therefore, could be offered by all community mental health teams (CMHTs) and physical health check services. SMI services should consider and assess sleep and physical activity/exercise issues and promote healthy effective sleep and physical activity/exercise within a recovery focused practice.

